# Combined analysis of the transcriptome and proteome of *Eucommia ulmoides* Oliv. (Duzhong) in response to *Fusarium oxysporum*

**DOI:** 10.3389/fchem.2022.1053227

**Published:** 2022-10-13

**Authors:** Yingxia Lu, Xuan Dong, Xiaozhen Huang, De-gang Zhao, Yichen Zhao, Lei Peng

**Affiliations:** ^1^ College of Tea Sciences, Guizhou University, Guiyang, China; ^2^ The Key Laboratory of Plant Resources Conservation and Germplasm Innovation in Mountainous Region (Ministry of Education), Guiyang, China; ^3^ Guizhou Academy of Agricultural Science, Guiyang, China

**Keywords:** transcriptome, proteome, *Eucommia ulmoides*, *Fusarium oxysporum*, endoplasmic reticulum

## Abstract

*Eucommia ulmoides* Oliv. (Duzhong), a valued traditional herbal medicine in China, is rich in antibacterial proteins and is effective against a variety of plant pathogens. *Fusarium oxysporum* is a pathogenic fungus that infects plant roots, resulting in the death of the plant. In this study, transcriptomic and proteomic analyses were used to explore the molecular mechanism of *E. ulmoides* counteracts *F. oxysporum* infection. Transcriptomic analysis at 24, 48, 72, and 96 h after inoculation identified 17, 591, 1,205, and 625 differentially expressed genes (DEGs), while proteomics identified were 66, 138, 148, 234 differentially expressed proteins (DEPs). Meanwhile, GO and KEGG enrichment analyses of the DEGs and DEPs showed that they were mainly associated with endoplasmic reticulum (ER), fructose and mannose metabolism, protein processing in the ER, type II diabetes mellitus, the ribosome, antigen processing and presentation, and the phagosome. In addition, proteome and transcriptome association analysis and RT-qPCR showed that the response of *E. ulmoides* to *F. oxysporum* was likely related to the unfolded protein response (UPR) of the ER pathway. In conclusion, our study provided a theoretical basis for the control of *F. oxysporum*.

## Introduction

*Eucommia ulmoides* Oliv. (Duzhong) is a traditional Chinese herbal medicine and is recorded in the ancient pharmacy classics, Shen Nong’s Herbal Classic and Compendium of Materia Medical (Anderson, 1982). It is the only existing species of the family Eucommiaceae (Tokumoto et al., 2016). The leaves and bark of *E. ulmoides* have been listed in the Chinese Pharmacopoeia (Qian et al., 2010), and its medicinal components have attracted extensive attention. It has been used to treat hypertension, hyperlipidemia, obesity, diabetes, inflammation, and other conditions. In addition, it also has antiviral, antibacterial, antioxidant, antitumor, and other activities (Qian et al., 2010; Fujiwara et al., 2016; Hussain et al., 2017; Cho et al., 2018; Lee et al., 2018).

*Fusarium oxysporum* is a widespread plant pathogenic fungus, belonging to the imperfect fungi. It is a soil-borne parasite and produces large numbers of sickle-shaped conidia through asexual reproduction after infecting the plant (Gordon and Martyn, 1997; Michielse and Rep, 2010). It infects the plant roots and causes deterioration of the vascular bundles, damages the functioning of the ducts, and may cause the death of the plant through damage at all stages of the plant growth (Pietro et al., 2010). *Fusarium* infection affects a large number of crops with important economic value, such as the Musaceae, Rosaceae, Cucurbitaceae, Brassicaceae, Solanaceae, and Fabaceae (Gordon and Martyn, 1997; Edel-Hermann and Lecomte, 2019), which has brought serious losses to agricultural production. Our laboratory’s previous research found that the total protein extracted from *E. ulmoides* had a significant inhibitory effect on *F. oxysporum* (Liu et al., 2007). However, the molecular mechanism of *E. ulmoides* counteracts *F. oxysporum* infection was not clear yet.

With the development of high-throughput sequencing technologies, “omics” analyses have been widely applied to the study of plant-microbe interactions, especially in the study of plant responses to pathogen stress. The transcriptome represents the sum of all transcripts produced by a particular tissue or cell of a species at a specific time (Guo et al., 2021). Transcriptome sequencing can determine the expression of all genes during a particular period, which provides an important reference for gene transcription (Wilhelm and Landry, 2009). Transcriptome sequencing has been used extensively to study biotic and abiotic stresses in plants, and the technology can provide a comprehensive picture of gene expression in target biological tissues under specific stress conditions. Comparative transcriptome analysis can identify changes in the expression of genes associated with specific physiological or pathological conditions (Shi et al., 2020). Proteomics is used to identify and quantify the overall protein content of cells, tissues, or organisms. It is an effective complement to other omics analyses, such as genomics and transcriptomics. It provides a useful basis for elucidating the structure and function of proteins, resolving cellular signaling pathways (Wasinger et al., 1995; Mann et al., 2001; Monti et al., 2005), and understanding pathogenic mechanisms (Saleh et al., 2019).

With the development of biological information, in this study, we explore to study the molecular mechanism of *E. ulmoides* in response to *F. oxysporum* using transcriptome and proteome methods. This study will provide a theoretical basis for the control of *F. oxysporum*.

## Materials and methods

### Materials

The seeds of *E. ulmoides* were collected from *E. ulmoides* trees that had been established for over 10 years in the Key Laboratory of Mountain Plant Resource Protection and Germplasm Innovation, Institute of Agricultural Bioengineering, Guizhou University. *F. oxysporum* was isolated from *Passiflora edulis* Sims according to the method of Sarwar et al. (2005) showing characteristic symptoms of wilt and identified by the Tea Biotechnology Laboratory of Tea College of Guizhou University. Plants of *P. edulis* Sims showing characteristic symptoms of wilt were collected with fibrous tertiary roots on the basis of visual observations and brought in the laboratory for isolation of *F. oxysporum*.

### Sample preparation

The peels of the mature seeds of *E. ulmoides* were removed and the seeds surfaces were thoroughly cleaned using sterile water. Then the seeds were soaked in sterile water for 12 h and rinsed with tap water. After disinfection with 75% alcohol for 60 s, the seeds were washed several times with sterile water, followed by soaking in NaClO solution with 10% available chlorine for 15 min. After several further washes with sterile water, the seeds were dried on sterile water-absorbing paper. The seed coat was then cut using a scalpel and inoculated on solid Murashige and Skoog (MS) basal medium with vitamins (cultured in a glass incubator containing 50 ml MS medium with a diameter of 5.5 cm and a height of 12 cm) and cultured under a light/dark cycle of 16/8 h at 25°C. After 15–20 days when the cotyledons were fully expanded, the roots of *E. ulmoides* seedlings with 2-4 true leaves were inoculated with *F. ulmoides*. The inoculated seedlings were cultured in dark at 25°C. After inoculation for 0, 24, 48, 72, and 96 h, the naked eye fungal infection and necrotic part were removed and the samples for subsequent experiments were collected from the tissue 1 cm above the removed tissue. Then, the collected samples were snap-frozen in liquid nitrogen and stored at -80°C.

### RNA extraction and transcriptome sequencing

The collected samples (100 mg) were ground with liquid nitrogen. Total RNA was extracted using the Plant RNA Extraction Kit (ComWin Biotech Co., Ltd., Beijing, China) according to the supplied method, and three replicates were prepared for each treatment. Poly-A tailed mRNA was enriched using Oligo dT magnetic beads. The RNA was then fragmented and reverse-transcribed using random N6 primers. After synthesis of double-stranded DNA, the ends were flattened and phosphorylated at the 5′ end, while the 3′ end had a protruding “A” sticky end. The ligated product was amplified by PCR with specific primers (forward primer: 5′-GAA​CGA​CAT​GGC​TAC​GA-3′ and reverse primer: 5′-TGT​GAG​CCA​AGG​AGT​TG-3′), the PCR product was heat-denatured to single-stranded DNA and then cyclized with a bridge primer to obtain a single-stranded circular DNA library and sequenced using DNBSEQ platform.

The raw sequencing data contains reads with low quality, splice contamination, and high contents of unknown base N. These reads need to be removed before data analysis to ensure the reliability of the results. The filtering software SOAPnuke version 1.4.0 was used for analysis and filtering was performed using Trimmomatic v0.36 to remove the reads containing the connector, those with unknown base N contents greater than 5%, and low-quality reads with mass values of less than 10 accounting for more than 20% of the total base number of the reads. The clean reads were then used for subsequent analysis and were mapped to the Eucommia reference genome (https://www.ncbi.nlm.nih.gov/bioproject/?term=PRJNA357336) (Chen et al., 2022) using HISAT 2.1.0 (Kim et al., 2015). The clean reads were also compared with reference gene sequences using Bowtie 22.2.5 (Langmead and Salzberg, 2012) and RSEM 1.2.8 (Li and Dewey, 2011) was used to calculate the expression levels of the genes and transcripts. All the programs were used with default parameters. The data that support the findings of this study have been deposited into CNGB Sequence Archive (CNSA) of China National GeneBank DataBase (CNGBdb) with accession number of CNP0003469.

### Protein extraction and mass spectrometry analysis

The total protein of *E. ulmoides* was extracted according to the method of Yu et al. (2018) and analyzed by Nano LC-1DTM plus system (Eksigent, Dublin, CA, United States) combined with Triple TOF5600 MS (Foster City, CA, United States).

Max Quant version 1.5.2.8 (Cox et al., 2011) was used for processing the LC-MS/MS data. The *E. ulmoides* protein database downloaded from NCBI was used for MS/MS identification. The results of the Max Quant analysis, including the initial search for the precursor mass with a tolerance of 20 μl/ml, were used for mass recalibration (Cox and Mann, 2008). The false discovery rate (FDR) for peptide and protein identification was set at 0.01. Label-free quantification with a minimum of two ratio counts was used for normalization and the sum of two ratio counts was used to determine the normalized protein levels for comparison between samples (Cox and Mann, 2008). The IBAQ algorithm was used to calculate the absolute abundance of different proteins in a single sample (Luber et al., 2010), and the proteins were filtered to eliminate common contaminants and the interference of opposite databases. The data that support the findings of this study have been deposited into CNGB Sequence Archive (CNSA) of China National GeneBank DataBase (CNGBdb) with accession number of CNP0003469.

### Functional annotation and enrichment analysis

To annotate unigenes which were assembled by Stringtie, sequences were searched by BLASTx against the NCBI non-redundant protein (nr) database and other databases, including the Swiss-Prot protein database, the Kyoto Encyclopedia of Genes and Genomes (KEGG) and the Gene Ontology (GO) databases. KEGG enrichment scatter plots and GO enrichment bar plots were constructed using the OmicStudio tools at https://www.omicstudio.cn/tool.

### Identiﬁcation of differentially expressed genes and differentially expressed proteins

The DESeq2 R package (https://genomebiology.biomedcentral.com/articles/10.1186/s13059-014-0550-8, accessed on November 2020) was used to analyses the differential expression between groups; it uses a model based on the negative binomial distribution to provide statistical routines for determining differential expressions in digital gene expression data. The resulting *p*-values were adjusted by using Benjamini and Hochberg’s approach to control the false discovery rate (FDR < 0.01). Genes with an adjusted *p* value < 0.01 by DESeq2 were assigned as differentially expressed genes (DEGs). The protein differences between two groups were analyzed by *t*-tests. Proteins with *p* values ≤ 0.01 were considered to be differentially expressed proteins (DEPs).

### Protein-mRNA correlation analysis

To correlate transcript and protein expression profiles, accession numbers from the proteome were extracted and compared with the annotated RNA-Seq libraries. A protein-mRNA correlation analysis was performed using the regularized-logarithm transformation (rlog) value of the spectral counts and the normalized log2 probe intensity for mRNAs. Briefly, we calculated the global Spearman correlation coefficient, rho, for protein-mRNA pairs within all DEPs and DEGs, similar regulatory patterns of DEPs and DEGs and opposite regulatory patterns of DEPs and DEGs, respectively. Adjusted *p* values based on the analysis of DEPs and DEGs were computed by the Benjamini-Hochberg procedure (Benjamini and Hochberg, 1995). The KEGG enrichment analysis and Advanced Heatmap Plots were performed using the OmicStudio tools by R version 3.6.3 (https://www.omicstudio.cn).

### Analysis of the relative gene expression

Total RNA was extracted from powdered *E. ulmoides* samples using the Plant RNA Extraction Kit (ComWin Biotech Co., Ltd., Beijing, China). The RNA was reverse-transcribed using a Goldenstar™ RT6 cDNA Synthesis Kit (Tsingke Biotechnology, Beijing, China) according to the manufacturer’s instructions. Real-time quantitative polymerase chain reaction (RT-qPCR) was performed using the reaction sample (10 μl) with SoFast EvaGreen^®^ Supermix (Bio-Rad, Hercules, CA, United States) and the RT-PCR assay was completed using the CFX ConnectTM Real-Time PCR System (Bio-Rad, Hercules, CA, United States), each sample was run in triplicate with appropriate negative controls, under the same condition used in previous studies: 95°C for 3 min, then 39 cycles of 95°C for 10 s, 65°C for 20 s, and 72°C for 10 s. Fluorescence data were collected at the end of the second step and, following cycling, the melting curve was determined in the range of 65–95°C with an increment of 0.05°C/s. Gene expression was normalized using elongation factor-1 alpha as an internal control. The specific primers used in RT-qPCR were designed by the RNA Folding Form (http://www.unafold.org/mfold/applications/rna-folding-form.php) and Primer Quest™ Tool (https://sg.idtdna.com/pages/tools/primerquest) and listed in Table 1. The 2^−∆∆Ct^ method was used to calculate the relative expression levels. Each treatment was analyzed for three times.

**TABLE 1:** List of primers used in this study.

No.	Gene ID	Annotation names	Forward primer (5′-3′)	Reverse primers (5′-3′)
1	GWHTAAAL010245	Elongation factor-1 alpha	TGA​TTG​AGA​GGT​CCA​CAA​ACC	CCA​ATC​TTG​TAG​ACA​TCC​TGG​AG
2	GWHGAAAL008628	Translation initiation factor eIF-2B subunit alpha	CAA​CGT​GTT​GCT​CGG​AAA​AG	GGT​GAA​CCT​TCA​TTC​GTG​GG
3	GWHGAAAL012002	BCL2-associated athanogene 1	AGG​AAA​TGG​GAA​TAA​GGT​GGC	AGC​ATG​TGA​TTC​TCC​ATC​GG
4	GWHPAAAL009648	Endoplasmic reticulum chaperone BiP	GGT​CAC​ATT​TGA​AGT​CGA​TGC	TCA​ATC​TCC​TCT​TGG​CTC​AAC
5	GWHGAAAL005471	HSP20 family protein	TTC​CCT​CTC​CTA​TCC​TCT​TCC	CTG​CCT​CTG​TCC​TGA​TTA​TGA​G
6	GWHGAAAL020098	Heat shock 70 kDa protein 1/2/6/8	AGC​AGA​GAA​ATA​CAA​GTC​AGA​GG	TGG​CCT​GAT​CAA​TAG​CAT​CC
7	GWHGAAAL010874	Hypoxia up-regulated 1	GGT​TGA​ATT​CTA​CTG​GGC​TCC	CAG​ATG​TAG​GTA​TTG​AAG​ACG​AGG
8	GWHPAAAL013860	Hsp70-Hsp90	AGAAACCAGAGCGGCAAC	CTT​TGG​TAT​AAT​GCT​GAA​TCG​CTG
9	GWHPAAAL019641	Protein disulfide-isomerase A6	GTC​CTA​GTG​GAA​TTC​TAT​GCT​CC	GTG​TCG​GCA​TCA​ACA​TTG​G
10	GWHPAAAL025197	Calnexin	AAA​AGA​CGA​TTC​CCG​ACC​C	GCA​TCC​TCA​TCC​CAG​TCA​TC
11	GWHPAAAL008473	Calreticulin	GAA​GAG​GAT​GGA​ATA​TGG​AGA​GC	CTT​CAA​CAC​ATA​GAG​GTC​GGG
12	GWHGAAAL023146	Ubiquitin-conjugating enzyme E2	ATG​GCT​CCG​AGA​AAA​TAC​GTC	CAT​ACA​CCA​ATC​GCC​AAG​TTG
13	GWHGAAAL022038	F-box and WD-40 domain protein 1/11	ATC​CGA​CAG​CAA​GAA​CAT​CC	AAC​ACG​AAC​CTT​ACC​ATC​CTG

## Results

### Morphological observations

**FIGURE 1:**
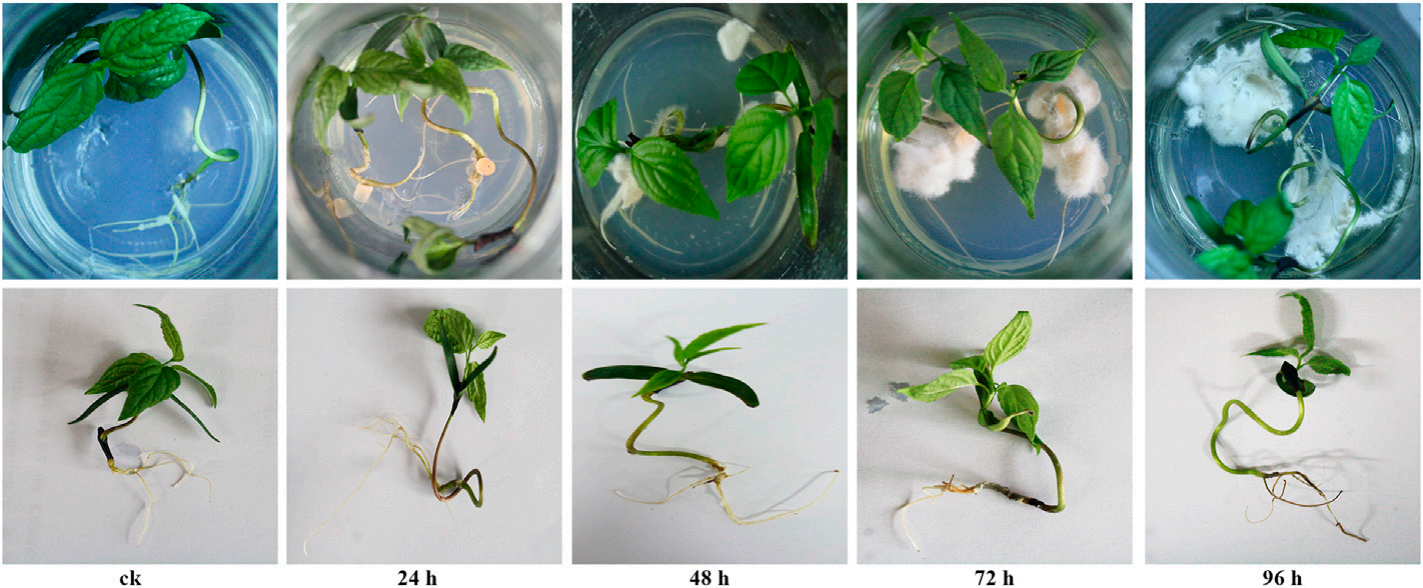
The roots morphology of *E. ulmoides* seedlings after 0, 24, 48, 72, and 96 h of inoculation with *F. oxysporum*.

The roots morphology of *E. ulmoides* seedlings inoculated with *F. oxysporum* was observed. As shown in Figure 1, after 24 h of inoculation, no mycelia were observed on the MS basal medium and the roots of *E. ulmoides* seedlings, while small numbers of new mycelia of *F. oxysporum* were visible after 48 h; no obvious lesions or necrosis were seen on the roots at either time. After 72 h of inoculation, large numbers of new mycelia of *F. oxysporum* were apparent, although there was no evidence of lesions or necrosis. After 96 h of inoculation, the root of *E. ulmoides* was eroded by *F. oxysporum*, with clear signs of necrosis and finger sheath-like detachments.

### RNA-Seq data analysis

A total of 15 samples (label as ck-1, ck-2, ck-3, 24h-1, 24h-2, 24h-3, 48h-1, 48h-2, 48h-3, 72h-1, 72h-2, 72h-3, 96h-1, 96h-2, 96h-3) were tested using the DNBSEQ platform. The quality indicators of the filtered reads are shown in Supplementary Table S1. The results showed that a total of 95.99 GB high-quality reads were obtained from the RNA-Seq transcriptome sequencing. Each sample had three independent biological replicates and each sample produced an average of 6.40 GB of data. The Q20 and Q30 of each sample were over 96.04% and 87.42%, respectively. The block diagram of the FPKM values for each sample was shown in Supplementary Figure S1, which showed that the data are accurate and could be used directly for further analysis. The average comparison rate between the samples and the genome was 68.49%. A total of 5177 new genes were predicted and the expression of 28,604 genes was detected, of which 23,478 were known genes and 5,126 were possibly new genes. A total of 57,545 new transcripts were detected, of which 15,439 belonged to new variable splicing subtypes of known protein-coding genes, 5,177 were transcripts of new protein-coding genes, and the remaining 36,929 were long non-coding RNAs.

### Bioinformatic analysis of differentially expressed genes

**FIGURE 2:**
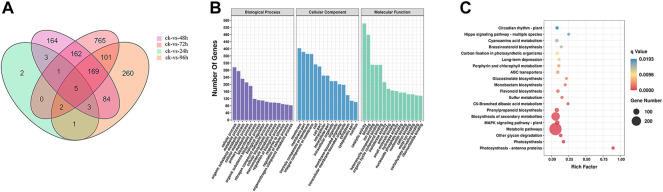
**(A)** Venn diagram of DEGs in *E. ulmoides* compared before (ck) with 24, 48, 72, and 96 h of inoculation with *F. oxysporum*, respectively. **(B)** GO analysis of DEGs in *E. ulmoides* between 0 and 72 h of inoculation with *F. oxysporum*. **(C)** KEGG enrichment analysis of DEGs in *E. ulmoides* between 0 and 72 h of inoculation with *F. oxysporum*.

After 72 h of inoculation with *F. oxysporum*, the Venn diagram (Figure 2A) showed that the greatest number (1205) of DEGs in *E. ulmoides* were present, and the DEGs are visually represented in the volcano plot (Supplementary Figure S2) with the numbers of up- and downregulated genes being 292 and 913, respectively. Meanwhile, after 72 h of inoculation, the annotation of genes using the GO database indicated that the total number of genes (TB) and the significantly DEGs between the inoculated and uninoculated treatments (TS) with *F. oxysporum* were 23,802 and 1,013, respectively. The top four DEGs annotations in the biological process (BP) category were found to be cellular process, metabolic process, organic substance metabolic process, and cellular metabolic process, with the numbers of S genes (the number of significantly DEGs annotated in the designated database) were 296, 274, 231, and 211, respectively. In the cellular components (CC) category, DEGs were enriched in the membrane, membrane part, intrinsic component of membrane, and integral component of membrane categories, with S gene numbers of 403, 382, 371, and 370, respectively. The CC categories of cell, cell part, intracellular, intracellular part, intracellular organelle, and organelle were also enriched, and the numbers of S genes were 312, 300, 249, 249, 218, and 218, respectively. In the molecular functions (MF) category, the DEGs were enriched in catalytic activity, binding, ion binding, heterocyclic compound binding, organic cyclic compound binding, and transferase activity. The numbers of genes were 542, 478, 310, 309, 309, 212, and 212, respectively (Figure 2B; Supplementary Figure S3). KEGG pathway enrichment (Figure 2C; Supplementary Figure S4) showed that there were 12,550 TB genes and 600 TS genes. DEGs were significantly enriched in metabolic pathways, biosynthesis of secondary metabolites, plant-pathogen interaction, MAPK signaling pathway-plant, fructose and mannose metabolism, phosphatidylinositol signaling system, and protein processing in ER, with the gene numbers of 288, 174, 56, 55, 12, 10, and 9, respectively.

### Proteome annotation analysis

After the component analysis (Supplementary Figure S5) and cluster analysis (Supplementary Figure S6) of the DEPs, the data from three biological replicates obtained after 0, 24, 48, 72, and 96 h of inoculation with *F. oxysporum* were analyzed, indicating that both the samples and the data were reliable and could be further analyzed. A total of 1,595 peptides were obtained by searching and analyzing the peptides in the total proteomic library of *E. ulmoides* obtained before and after inoculation with *F. oxysporum*. Overall, 1397, 1268, 1206, 1220, and 1166 peptides were obtained after 0, 24, 48, 72, and 96 h of inoculation, respectively.

**FIGURE 3:**
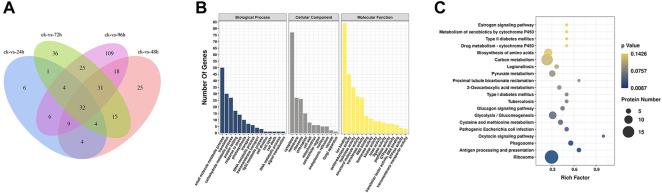
**(A)** Venn diagram of DEPs in *E. ulmoides* compared before (ck) with 24, 48, 72, and 96 h of inoculation with *F. oxysporum*, respectively. **(B)** GO analysis of DEPs in *E. ulmoides* between 0 and 72 h of inoculation with *F. oxysporum*. **(C)** KEGG enrichment analysis of DEPs in *E. ulmoides* between 0 and 72 h of inoculation with *F. oxysporum*.

The Venn diagram (Figure 3A) showed that the number of DEPs was highest at 96 h after inoculation with *F. oxysporum*. The DEPs were visualized using a volcano plot (Supplementary Figure S7), indicating that eight proteins were up-regulated and 226 were downregulated after 96 h of inoculation compared with their expression before inoculation. The GO database indicated that the significant DEPs were enriched in small-molecule metabolic process, translation, carbohydrate metabolic process, protein folding, response to stress, and other BP. The number of DEPs in these categories were 50, 30, 27, 17, and 14, respectively. In the CC category, DEPs were enriched in cytoplasm, membrane, ribosome, chloroplast, and chloroplast, with 77, 27, 26, and 15 proteins in the respective categories. In terms of MF, the numbers of DEPs enriched in ion binding, oxidoreductase activity, hydrolase activity, structural molecule activity were 84, 45, 35, 28, and 27, respectively (Figure 3B; Supplementary Figure S8). KEGG pathway analysis (Figure 3C; Supplementary Figure S9) showed the most significant enrichment in the ribosome, carbon metabolism, biosynthesis of amino acids, glycolysis/gluconeogenesis, pyruvate metabolism, protein processing in ER, phagosome, antigen processing and presentation, and type II diabetes categories. The number of DEPs in each of these pathways was 18, 15, 10, 9, 7, 6, 5, 4, and 2, respectively.

### Analysis of the association between the proteomic and transcriptomic data

**FIGURE 4:**
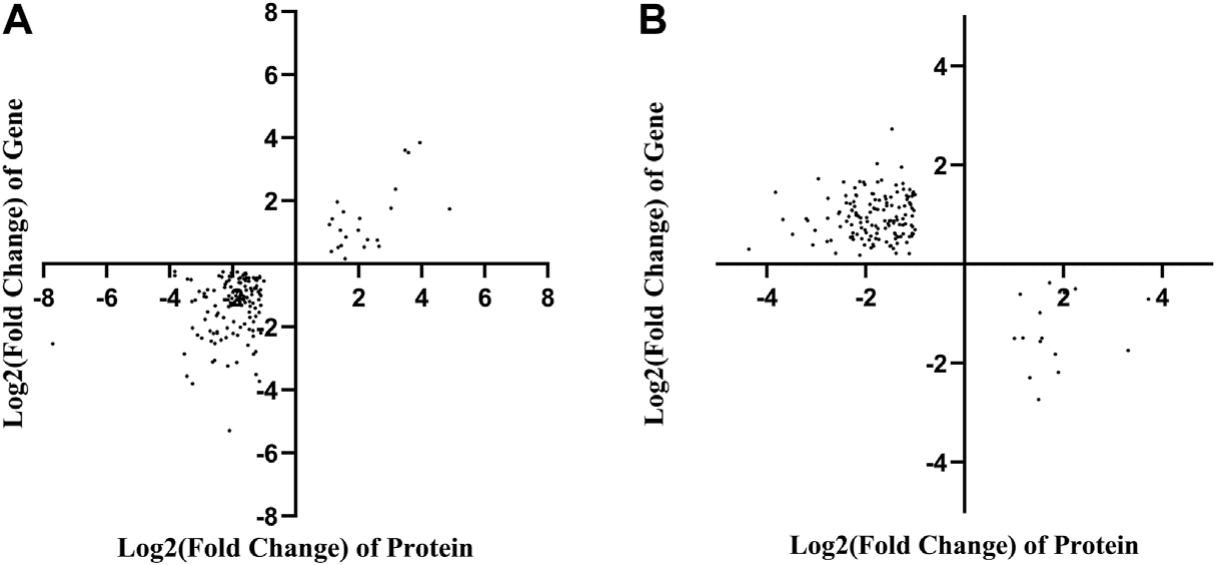
Correlation analysis based on the proteomics and transcriptomics. **(A)** Correlation analysis of differentially expressed proteins and genes with the same tendency of change. **(B)** The correlation analysis of significant differential expression of proteins and genes with opposite trends.

**FIGURE 5:**
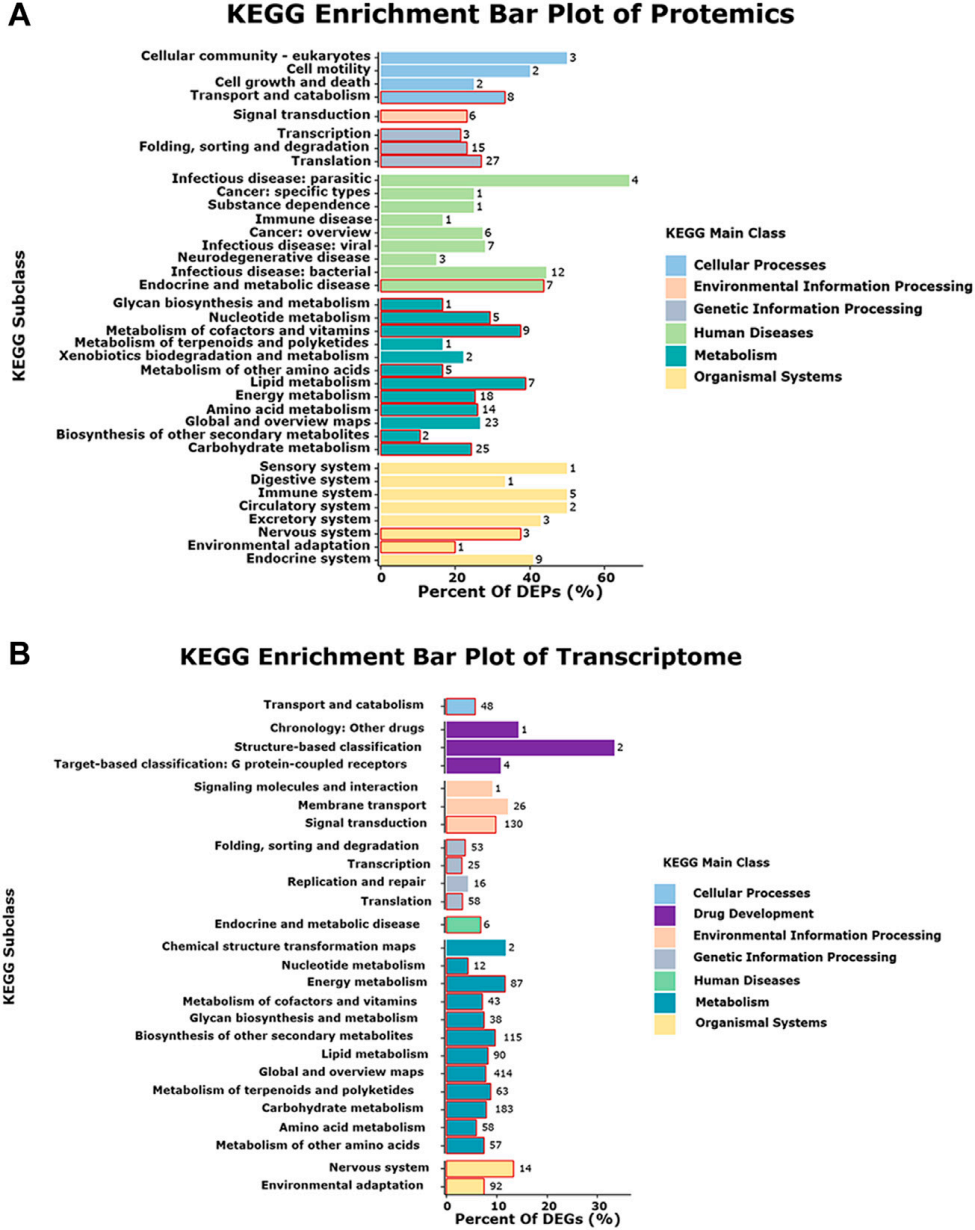
Pathway enriched in both the proteome and transcriptome. The red boxes represent in the pathways that are enriched in both protein and mRNA. **(A)** KEGG enrichment bar plot of Protemics. **(B)** KEGG enrichment bar plot of Transcriptome.

To correlate the transcript and protein expression profiles, the DEPs from the proteomic analysis were compared with the annotated RNA-Seq library. The results showed that 153 of the 326 proteins were expressed in the same pattern as their mRNAs, indicating that the expression of almost half of the proteins was regulated directly at the transcriptional level. Cluster analysis on these 326 DEGs and DEPs (Supplementary Figure S10) indicated that both DEGs and DEPs were reliable and could be further analyzed. Figure 4 showed 153 and 158 genes with similar and opposite regulatory patterns at the transcriptional and protein levels were analyzed for correlations, obtaining the pearson correlation coefficients (r) of 0.718 and -0.677, respectively. The red boxes in the KEGG pathways (Figure 5) highlight the pathways in which both DEPs and DEGs were common enriched in transcription, translation, and folding, sorting and degradation.

### RT-qPCR results

**FIGURE 6:**
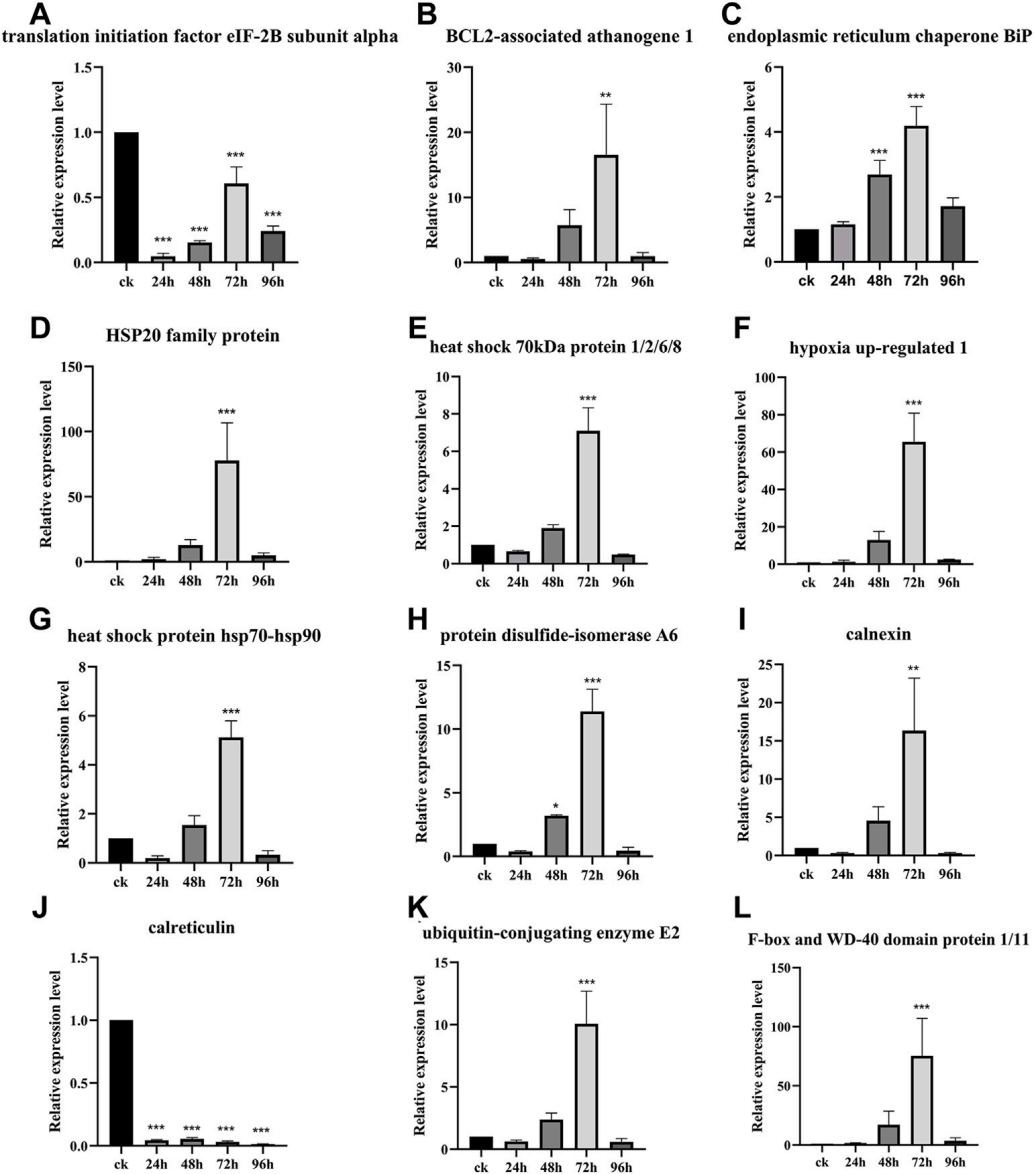
Gene relative expression analysis of the ER pathway-related genes. Data are shown as the mean of three biological replicates ± SD. The * represents significant difference (**p < 0.01, ***p < 0.001, Student’s *t*-test). **(A)** Translation initiation factor eIF-2B subunit alpha. **(B)** BCL2-associated athanogene 1. **(C)** Endoplasmic reticulum chaperone BiP. **(D)** HSP20 family protein. **(E)** Heat shock 70 kDa protein 1/2/6/8. **(F)** Hypoxia upregulated 1. **(G)** Heat shock protein hsp70-hsp-90. **(H)** Protein disulfide-isomerase A6. **(I)** Calnexin. **(J)** Calreticulin. **(K)** Ubiquitin-conjugating enzyme E2. **(L)** F-box and WD-40 domain protein 1/11.

The relative expression level of the DEPs and DEGs enriched in the ER pathway (ID: ko04141) were further analyzed using RT-qPCR method. The results showed that the relative expression level of translation initiation factor eIF-2B subunit alpha after 24, 48, 72, and 96 h of inoculation with *F. oxysporum* was 0.05, 0.15, 0.61, and 0.24 times lower than that of ck group (Figure 6A). The relative expression level of BCL2-associated athanogene 1 was 5.75 and 16.53 times higher at 48 and 72 h after inoculation, respectively, than ck group (Figure 6B). The ER contains at least three types of molecular chaperones of protein folding. The first of these includes the heat shock molecular chaperones such as HSP20, HSP70, and HSP90, the second includes the lectin molecular chaperones calcium-linked protein and calcium network protein, while the last category comprises enzymes related to protein processing and Ca^2+^ homeostasis, such as protein disulfide isomerase (PDI). The results of this study (Figures 6C–J) showed that, except for calreticulin, whose expression was suppressed after inoculation with *F. oxysporum*, the expression of all other molecular chaperones, including ER chaperone BiP, HSP20 family protein, heat shock 70 kDa protein 1/2/6/8, heat shock protein hsp70-hsp90, PDI A6, and calnexin, increased significantly at 72 h after inoculation, with the relative expression of hypoxia upregulated 1 increased 65.53 times more than before inoculation. During ER stress, the ER-associated degradation (ERAD) is activated at the transcriptional level to remove misfolded proteins, increasing their degradation by the ubiquitin-proteasome system. Figures 6K-L showed that the expression of the ubiquitin-binding enzyme E2 and ubiquitin-ligase F-box increased significantly at 72 h after inoculation, which were 10.07 and 75.26 times higher than those before inoculation, respectively.

## Discussion

*F. oxysporum* is an important pathogenic fungus, which can cause skin and eye lesions in mammals through infection of breaks in the skin, and also causes fusarium wilt in many crops through soil transmission, leading to serious economic losses in agricultural production. The control of *F. oxysporum* in agricultural production relies mainly on chemical fungicides which pose a major risk to the environment and food safety (Mukhtar, 2007; Jahanshir and Dzhalilov, 2010; Swarupa et al., 2014). It is, thus, important to investigate the antimicrobial mechanisms of traditional Chinese herbal medicine for the development of plant immune inducer (Dewen et al., 2017) and antimicrobial drugs with high efficacy and low toxicity. *E. ulmoides* is a traditional Chinese medicinal plant, recorded in The Classic of Herbal Medicine, that has proven antifungal property against *Botrytis cinerea* in our previous study (Liu et al., 2008).

In our present study, transcriptomic and proteomic analyses results showed that the KEGG pathway mainly concentrated in protein processing in ER, fructose and mannose metabolism, phosphatidylinositol signaling system, ribosome, protein processing in ER, phagosome, and antigen processing and presentation. Some previous studies (Malhotra and Kaufman, 2007; Wen and Wei, 2015; Ma, 2019; Zhang and Wei, 1997) had demonstrated that these pathways were related to the ER and ER misfolding. Therefore, our results demonstrated that *E. ulmoides* responds to *F. oxysporum* infection by mechanisms related to ER. The ER is an important organelle in the secretion pathway of eukaryotes and participates in the coordination of a variety of cellular activities such as protein processing and calcium homeostasis. It is also one of the important hubs for plant responses to various stresses (Martinez and Chrispeels, 2003; Helenius and Aebi, 2004; Kamauchi et al., 2005; Chen and Brandizzi, 2013; Doblas et al., 2013; Howell, 2013; Mishiba et al., 2013; Pollier et al., 2013; Cai et al., 2014; Williams et al., 2014). When plants are stressed by pathogenic infections, there is an accumulation of large numbers of unfolded or misfolded proteins in the ER lumen, leading to ER stress and adversely affecting the normal functioning of the organelle (Howell, 2013). If the protein accumulation exceeds the ER capacity, the cell responds by upregulating specific ER-associated genes and enzymes to restore homeostasis. This process is termed the ER stress response (Howell, 2013; Jing and Wang, 2020). Plant ER stress sensors can recognize ER stress and trigger the ER stress response, also called the unfolded protein response (UPR). The UPR has been shown to play an important role in interactions between plants and microorganisms (Wan and Jiang, 2016). The ER stress signaling pathway is closely related to plant immunity and represents an important part of the plant’s resistance to pathogen infection (Zhang et al., 2016; Qiang et al., 2021; Zhou et al., 2021). However, there is a lack of information on how plants respond to pathogen infection and activate ER stress-mediated immunity (ERSI).

The RT-qPCR results showed that the gene relative expression of ER chaperone protein BiP was significantly up-regulated after 48 and 72 h of inoculation with *F. oxysporum*. Upregulated expression of BiP is often considered to be a marker for ERS and UPR activation (Pobre et al., 2019). Meanwhile, the gene relative expression of translation initiation factor eIF-2B subunit alpha was inhibited after inoculation with *F. oxysporum*. It is possible that this could be the result of incorrect folding or wrong assembly of the protein, leading to binding by BiP to retain its unfolded state and prevent further misfolding. These reactions can lead to the phosphorylation of protein kinase R (PKR)-like ER kinase (PERK) protein dimer and cytoplasmic domain and eIF2. Phosphorylation of the latter at serine 51 blocks the binding of eIF2 to ATP and reduces the translation of its downstream mRNA (Harding et al., 2002; Ni et al., 2009). These reactions reduce the translation and synthesis of proteins, reduce the influx of new proteins to the ER, and thus prevent further increases in the numbers of unfolded proteins, thus lowering ERS (Harding et al., 2002; Ni et al., 2009). Combining the results of the analysis of BiP and eIF2α in this study with reported results, we speculate that the activation of the UPR in the *E. ulmoides* response to *F. oxysporum* is likely to be *via* the PERK/ATF4 of the ER pathway. This would prolong ERS and prevent the restoration of intracellular homeostasis and apoptosis would be induced by activation of the apoptosis regulator B lymphocyte 2 gene family (Marciniak et al., 2004). In this study, we also observed a significant increase in the expression of the BCL2-associated athanogene, further suggesting that the PERK/ATF4 of the ER pathway may play an important role in *E. ulmoides* response to *F. oxysporum*. At present, most of the studies on the UPR in plants have focused on IRE1-X box binding protein, transcriptional activator 6, and site-2 proteases (S2P) (Celli and Tsolis, 2015). However, the PERK/ATF4 of the ER pathway has only been studied in animals and has not been reported in plants (Zhang et al., 2021). Both the proteomic and transcriptomic association analysis and RT-qPCR verification indicated that the response of *E. ulmoides* to *F. oxysporum* is likely to be associated with the PERK/ATF4 of the ER pathway.

The UPR can coordinate transcriptional and translational changes in cells, up-regulate the expression of molecular chaperones involved in folding and stabilizing the proteins in the ER lumen, and induce the expression of stress proteins in the ER. In this study, the expression of ER molecular chaperones such as HSP20, HSP70, HSP90, protein disulfide isomerase (PDI), and calnexin (CNX) was significantly increased at 72 h after inoculation. It has been reported that HSP chaperones such as BIP and HSP90 can recognize misfolded proteins (Park and Seo, 2015; Usman et al., 2017; Ul Haq et al., 2019). The lectin molecular chaperones calnexin (CNX), calreticulin (CRT), and CRT are proteins related to protein processing and Ca^2+^ homeostasis, including PDI, CNX, and CRT, which could help localize unfolded proteins in the ER lumen to prevent unfolded/misfolded proteins from being exported. In ERS, the ERAD is activated at the transcriptional level to remove and degrade misfolded proteins. This process restores protein homeostasis (Harding et al., 1999; Balch et al., 2008). In addition, CNX links misfolded proteins through transmembrane domain interactions, activating protein degradation by the ubiquitin-proteasome system and signal transduction associated with phagocytosis, thus accomplishing the degradation of unfolded or misfolded proteins. The role of protein ubiquitination in plant growth and development and stress response has been widely recognized (Belknap and Garbarino, 1996). In this study E2 and F-box protein were significantly increased at 72 h after inoculation. These results suggest that molecular chaperones in the ER pathway (ko04141) are also involved in the response of *E. ulmoides* to *F. oxysporum*.

**FIGURE 7:**
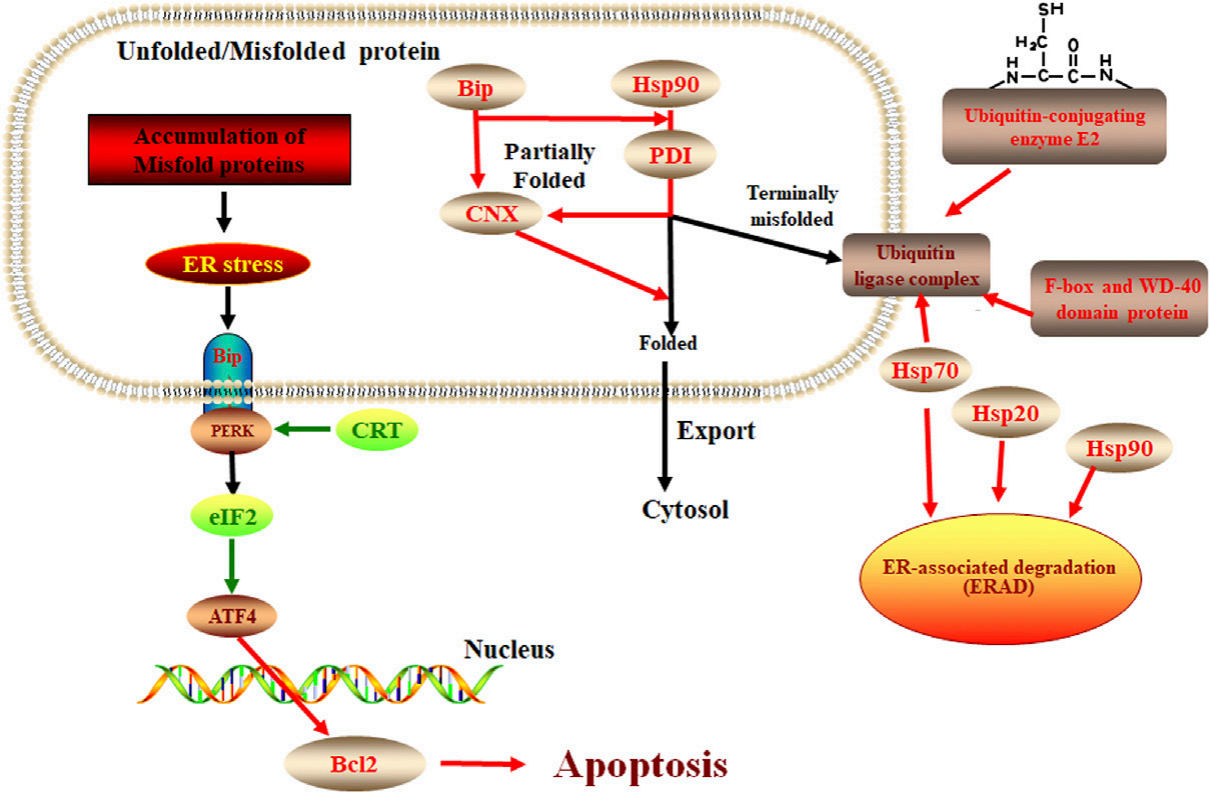
The protein processing in the ER pathway in *E. ulmoide* response to *F. oxysporum*. Red color represents up-accumulated genes or proteins in this pathway, while the green color represents down-accumulated.

ER stress response plays an important role in plant response to pathogen infection and other stresses, extensive evidence shows that the ER pathway contributes to plant immunity in various ways (Li et al., 2009; Nekrasov et al., 2009; Saijo et al., 2009). There is also evidence that the ER pathway is manipulated by microbes to lead to successful colonization. For example, the symbiotic fungus *Piriformospora indica* establishes successful symbiosis by inducing plant ER stress but inhibiting the adaptive UPR, which eventually results in ER-PCD mediated by vacuolar processing enzymes (VPEs) (Qiang et al., 2012). In our study, we found the response of *E. ulmoides* to *F. oxysporum* was likely related to the unfolded protein response of the ER pathway. Some studies have shown that the process of apoptosis is contrary to the expression of CRT (Xu et al., 2014). Our study showed that when *E. ulmoides* was infected by *F. oxysporum*, the increased expression of ER chaperone proteins makes the protein fold correctly and causes ubiquitination and ERAD reaction, while the inhibition of CRT and EIF2 expression increases the expression of BCL2 and causes apoptosis, so as to protect plants (Figure 7). In addition, we also observed that the relative expression of the hypoxia upregulated 1 gene of the HSP70 family and the F-box and WD-40 domain protein 1/11 gene of the ERAD were highest at 72 h after inoculation, being 65.53 and 75.26 times greater, respectively, than before inoculation. It is speculated that these two genes may be key to the *E. ulmoides* infection response in the activation of ER pathway.

## Conclusion

In this study, proteome and transcriptome association analysis and RT-qPCR results showed that the response of *E. ulmoides* to *F. oxysporum* was likely related to the ER pathway. In the future, we will study the genes functions in the ER pathway through model plant transgenic technology to further explore the role of these genes in the response of *E. ulmoides* to *F. oxysporum*.

## Data Availability

The datasets presented in this study can be found in online repositories. The names of the repository/repositories and accession number(s) can be found below: https://db.cngb.org/- CNP0003469.
