# Supplementary material for: Combined analysis of the transcriptome and proteome of Eucommia ulmoides Oliv. (Duzhong) in response to Fusarium oxysporum
Source: Front Chem. 2022 Oct 13;10:1053227. doi: 10.3389/fchem.2022.1053227 (PMC9606346; doi:10.3389/fchem.2022.1053227)
Supplement: Supplementary file 1 [file DataSheet1.docx]

**Supplementary Materials**

Table S1. Data filtering and quality statistics after filtering

| Sample | Total raw reads (M) | Total clean reads (M) | Total clean bases (Gb) | Clean reads Q20 (%) | Clean reads Q30 (%) | Clean reads ration (%) |
| --- | --- | --- | --- | --- | --- | --- |
| ck-1 | 43.3 | 42.77 | 6.42 | 96.63 | 88.98 | 98.76 |
| ck-2 | 43.3 | 42.87 | 6.43 | 96.69 | 89.16 | 99.01 |
| ck-3 | 43.3 | 42.73 | 6.41 | 96.54 | 88.77 | 98.67 |
| 24h-1 | 43.3 | 42.52 | 6.38 | 96.72 | 89.3 | 98.2 |
| 24h-2 | 43.3 | 42.46 | 6.37 | 96.83 | 89.56 | 98.04 |
| 24h-3 | 43.3 | 42.47 | 6.37 | 96.43 | 88.48 | 98.06 |
| 48h-1 | 43.3 | 42.95 | 6.44 | 96.06 | 87.42 | 99.18 |
| 48h-2 | 43.3 | 42.7 | 6.41 | 96.59 | 88.91 | 98.61 |
| 48h-3 | 43.3 | 42.87 | 6.43 | 96.63 | 89 | 99 |
| 72h-1 | 43.3 | 42.75 | 6.41 | 96.04 | 87.48 | 98.72 |
| 72h-2 | 43.3 | 42.57 | 6.83 | 96.76 | 89.37 | 98.29 |
| 72h-3 | 43.3 | 42.62 | 6.39 | 96.23 | 88.03 | 98.42 |
| 96h-1 | 43.3 | 42.33 | 6.35 | 96.22 | 88.03 | 97.76 |
| 96h-2 | 43.3 | 42.74 | 6.41 | 96.27 | 88.05 | 98.7 |
| 96h-3 | 43.3 | 42.61 | 6.39 | 96.89 | 89.22 | 98.41 |


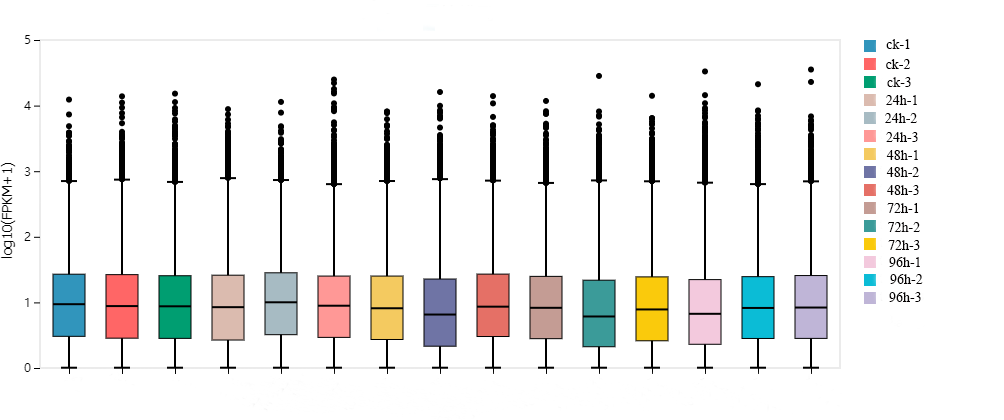


Figure S1. Expression of FPKM genes after 0, 24, 48, 72 and 96 h of inoculation with *F. oxysporum*.


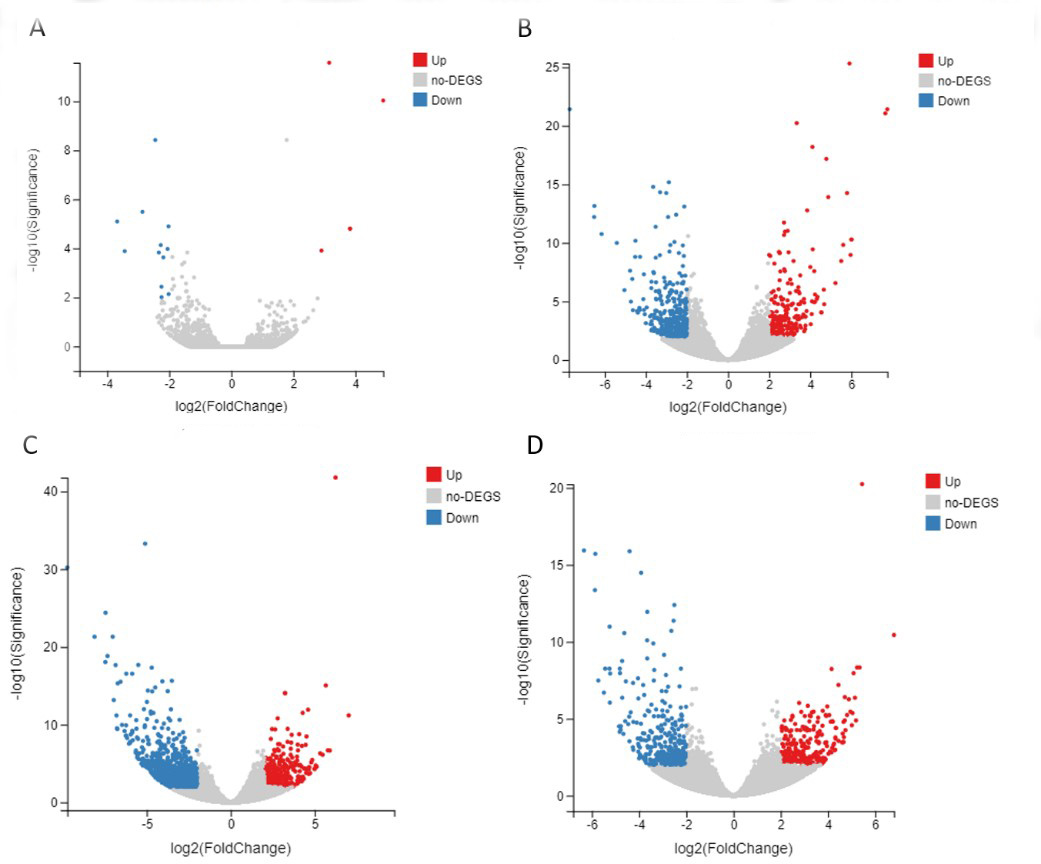


Figure S2. The volcano map of DEGs in *E. ulmoides* of 0 h (ck) vs 24 h (**A**), 0 h (ck) vs 48 h (**B**), 0 h (ck) vs 72 h (**C**), and 0 h (ck) vs 96 h (**D**), respectively.


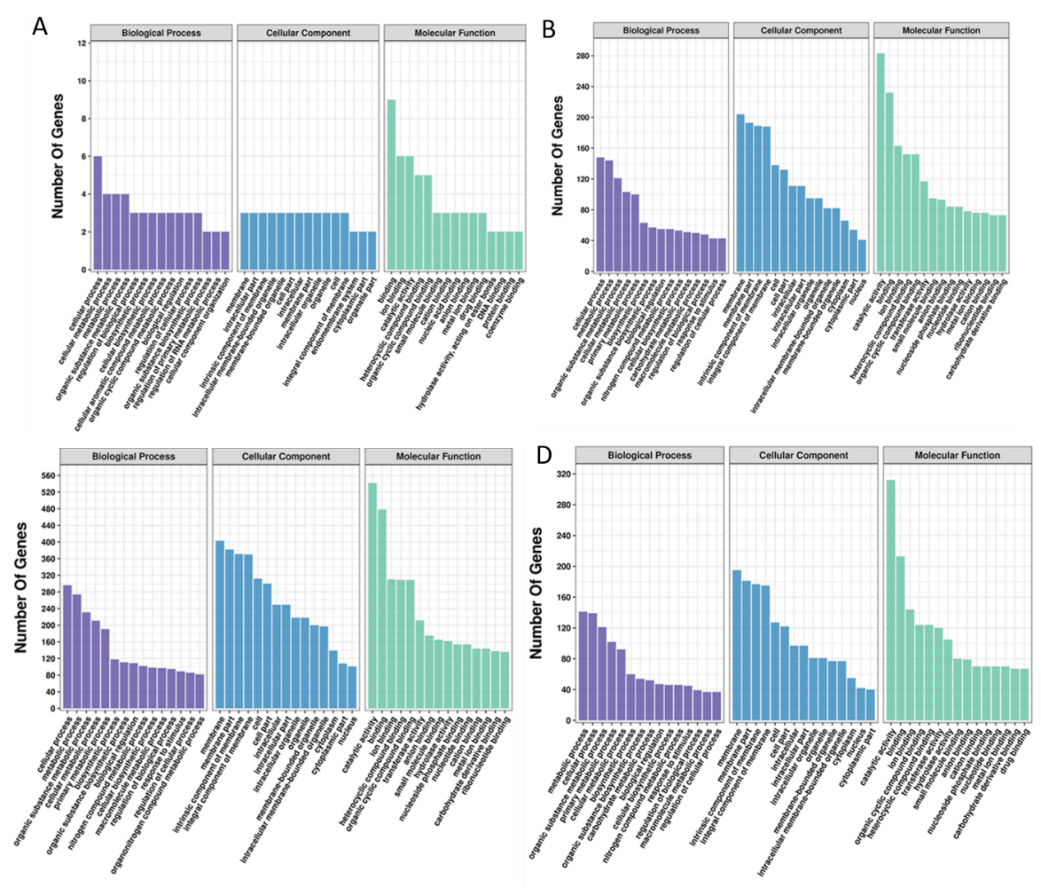


Figure S3. GO analysis of DEGs in *E. ulmoides* of 0 h (ck) vs 24 h (**A**), 0 h (ck) vs 48 h (**B**), 0 h (ck) vs 72 h (**C**), and 0 h (ck) vs 96 h (**D**), respectively.


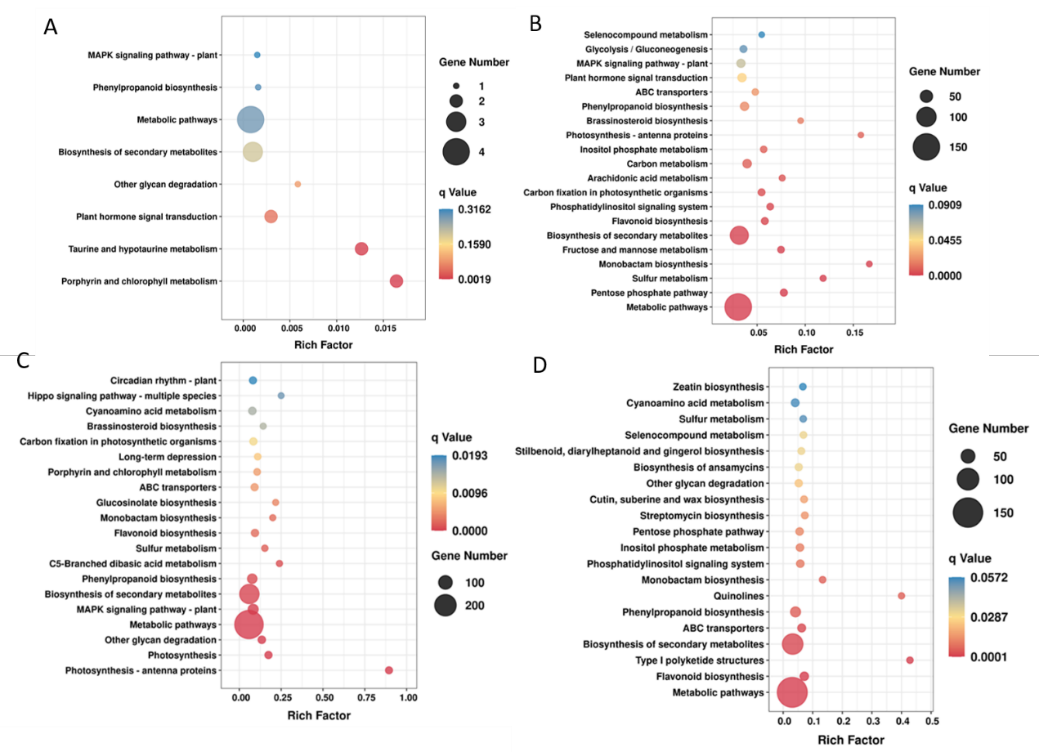


Figure S4. KEGG enrichment of DEGs in *E. ulmoides* of 0 h (ck) vs 24 h (**A**), 0 h (ck) vs 48 h (**B**), 0 h (ck) vs 72 h (**C**), and 0 h (ck) vs 96 h (**D**), respectively.


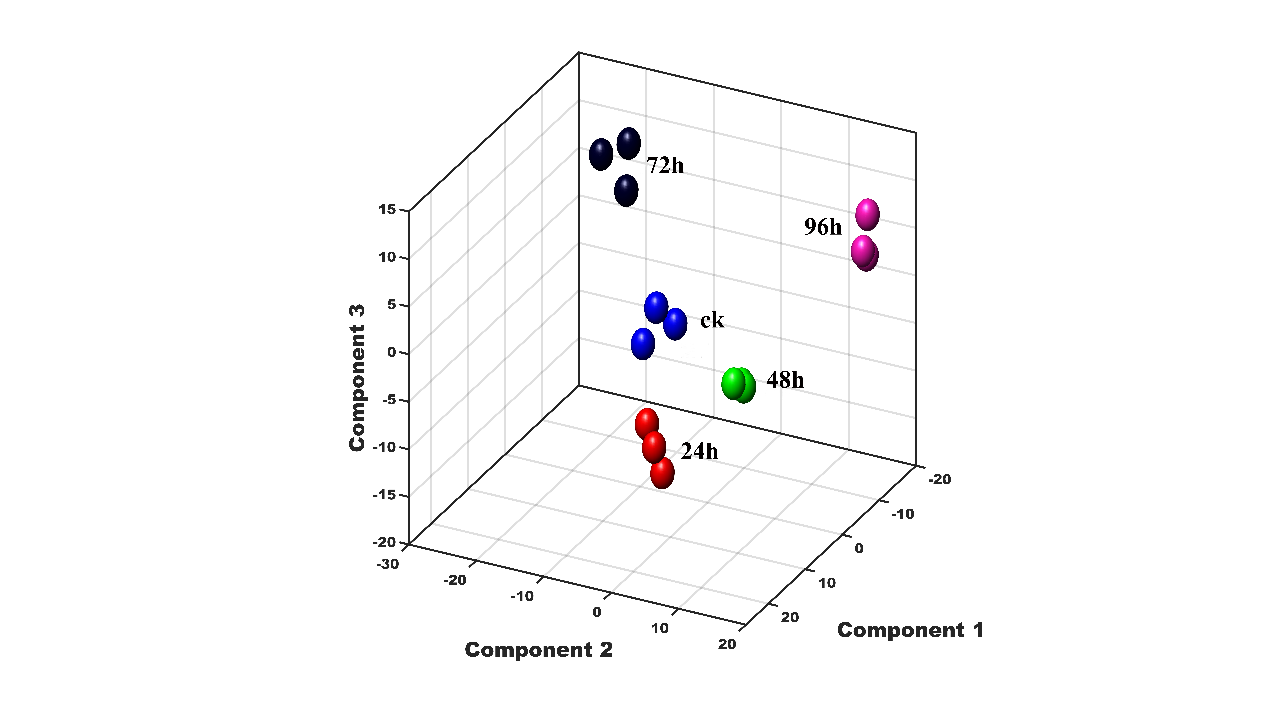


Figure S5. Analysis of differential protein compositions.


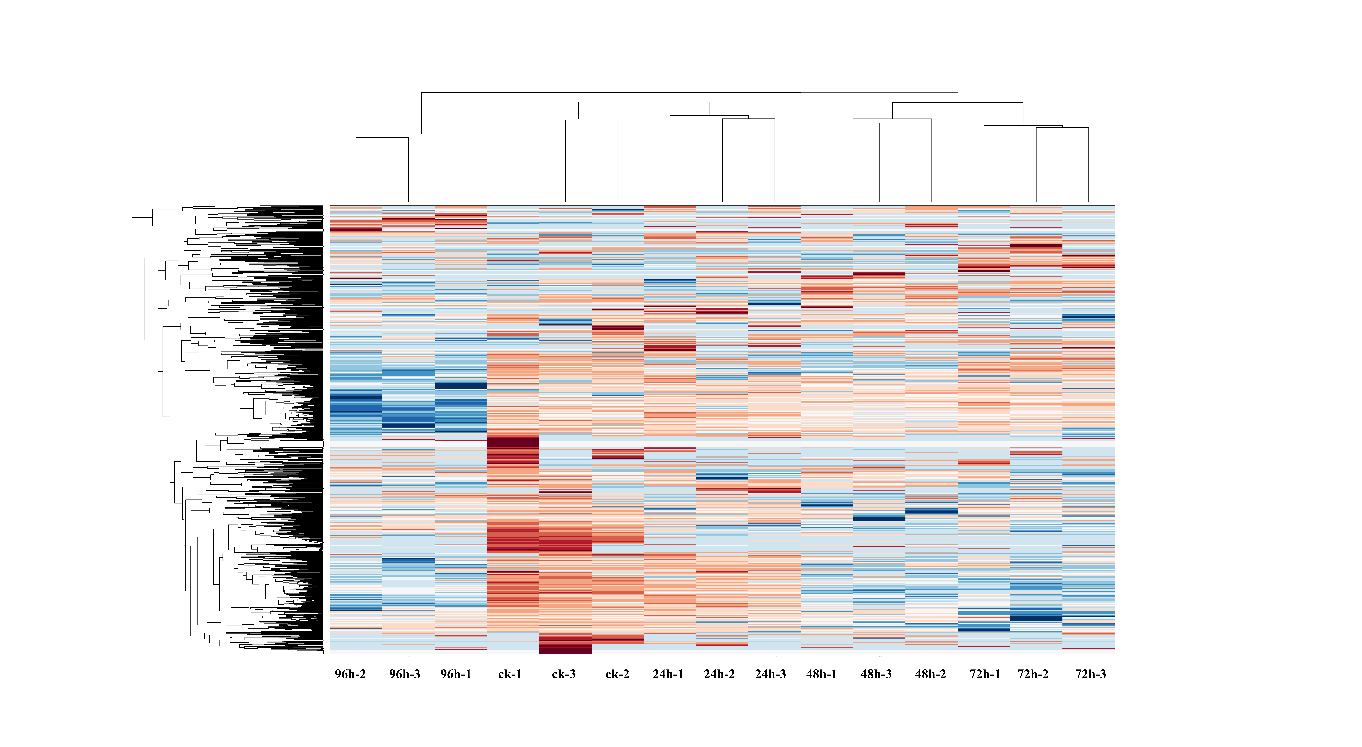


Figure S6. Cluster analysis of differentially expressed proteins


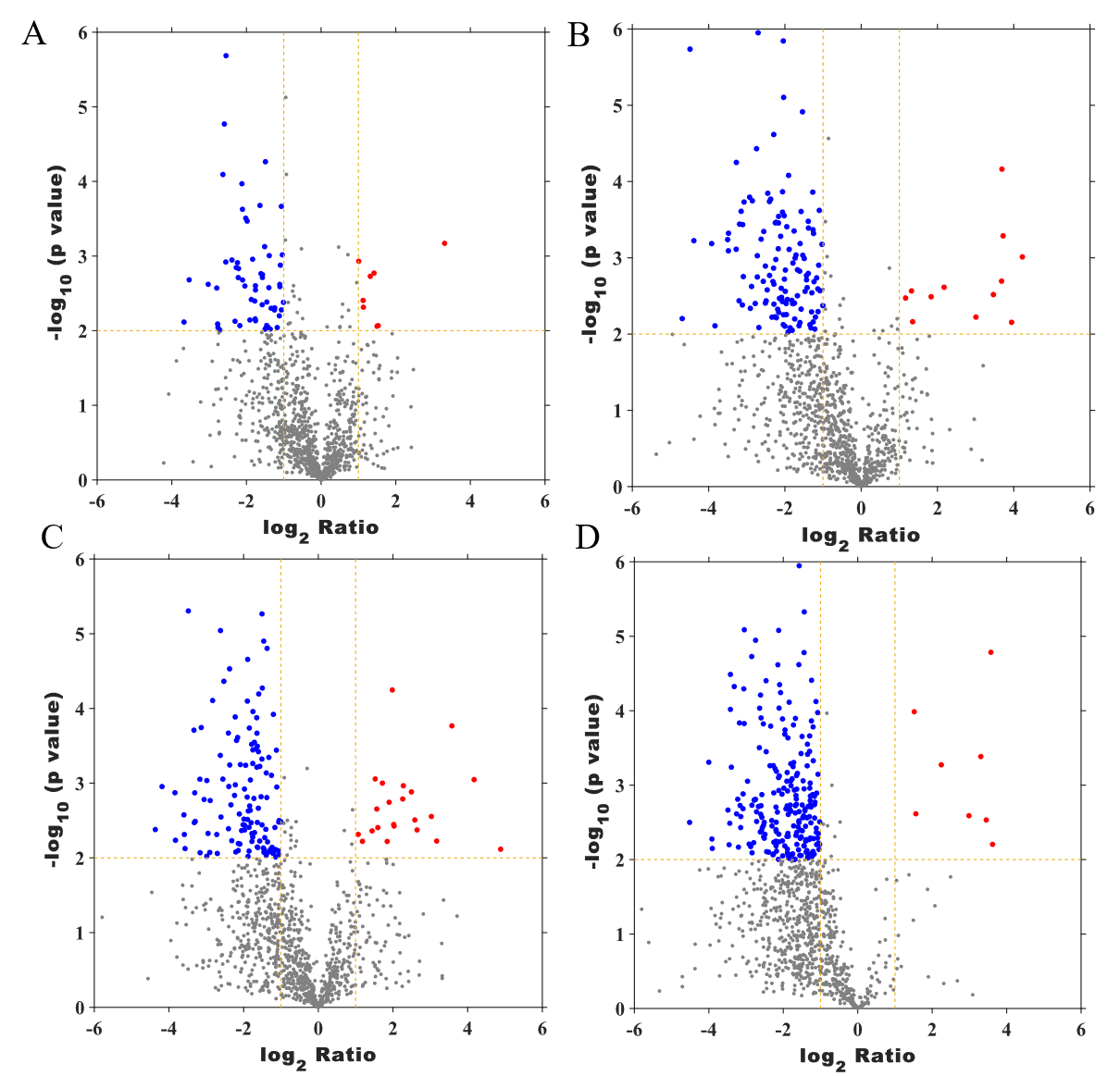


Figure S7. The volcano map of DEPs in *E. ulmoides* of 0 h (ck) vs 24 h (**A**), 0 h (ck) vs 48 h (**B**), 0 h (ck) vs 72 h (**C**), and 0 h (ck) vs 96 h (**D**), respectively.


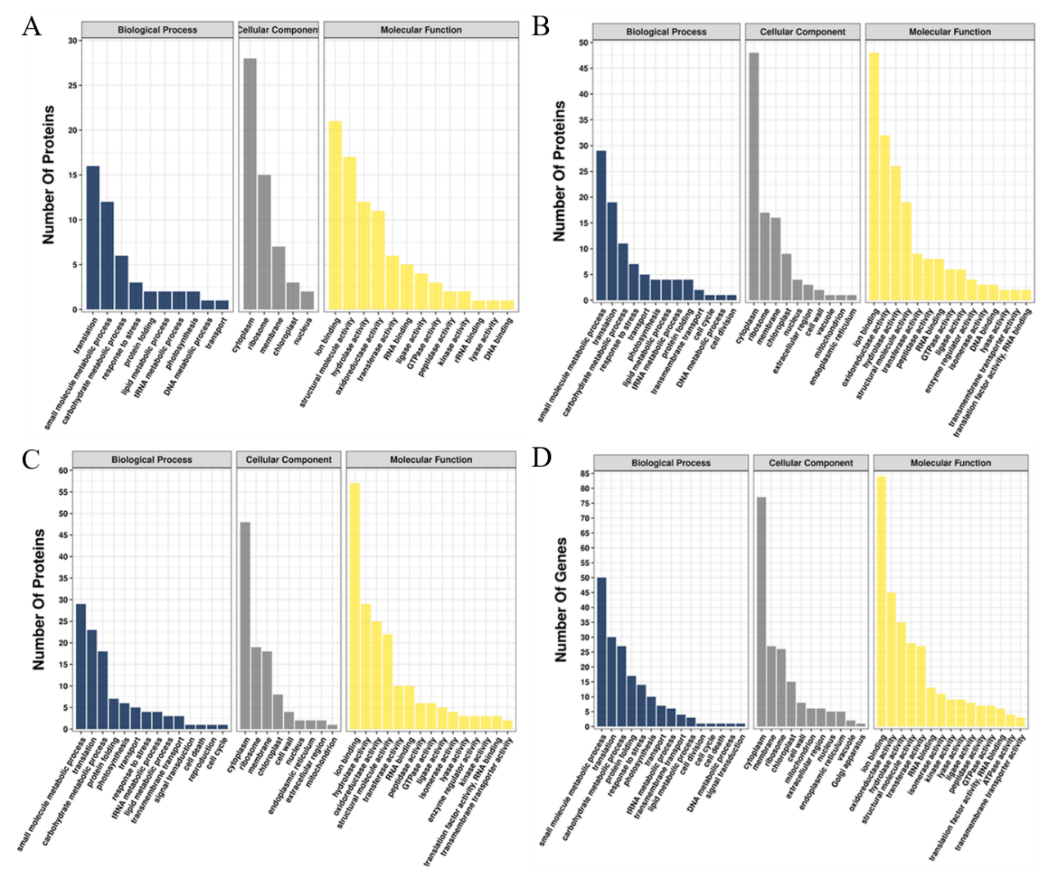


Figure S8. Go analysis of DEPs in *E. ulmoides* of 0 h (ck) vs 24 h (**A**), 0 h (ck) vs 48 h (**B**), 0 h (ck) vs 72 h (**C**), and 0 h (ck) vs 96 h (**D**), respectively.

**
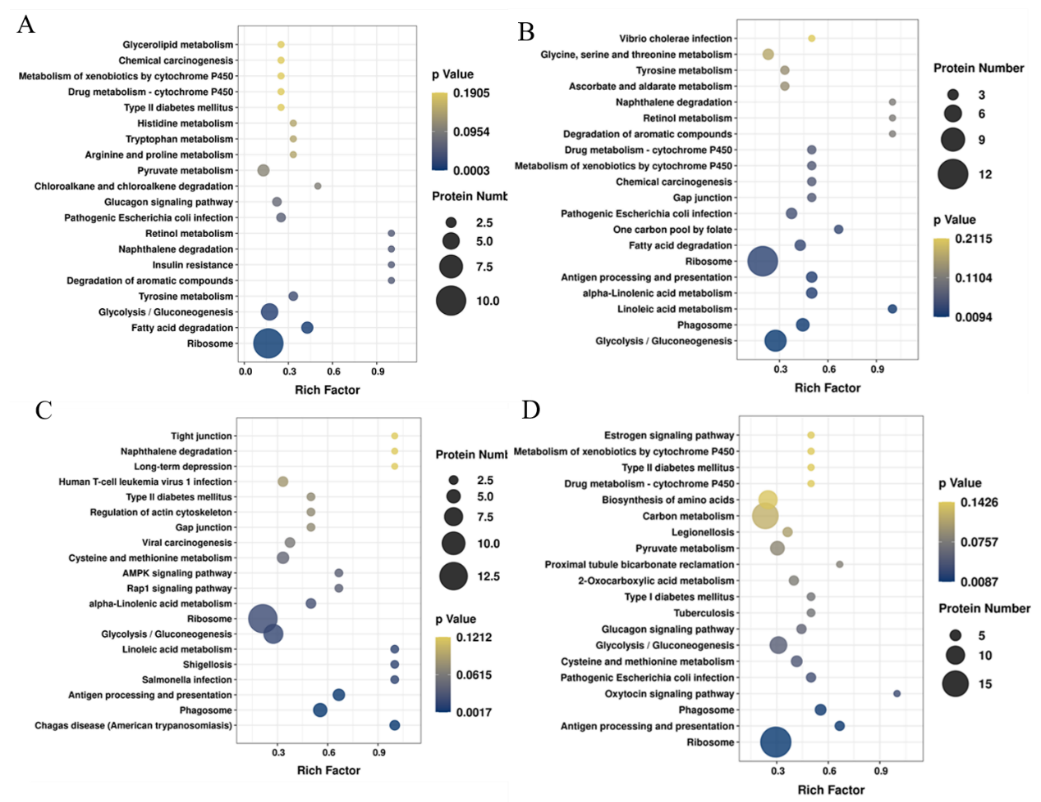
**

Figure S9. KEGG enrichment of DEPs in *E. ulmoides* of 0 h (ck) vs 24 h (**A**), 0 h (ck) vs 48 h (**B**), 0 h (ck) vs 72 h (**C**), and 0 h (ck) vs 96 h (**D**), respectively.


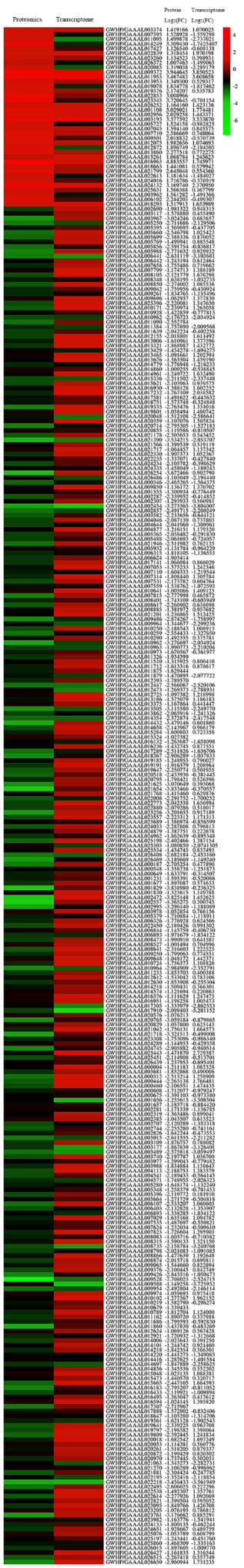


Figure S10. Cluster analysis of the expression patterns of 326 DEGs and DEPs. FC: Fold change. Red color indicates up-regulation and green color indicates down-regulation.
